# Assessing the Efficacy of Adjustable Moving Averages Using ASEAN-5 Currencies

**DOI:** 10.1371/journal.pone.0160931

**Published:** 2016-08-30

**Authors:** Jacinta Chan Phooi M’ng, Rozaimah Zainudin

**Affiliations:** Faculty of Business and Accountancy, University of Malaya, Kuala Lumpur, Wilayah Persekutuan, Malaysia; Universidad Veracruzana, MEXICO

## Abstract

The objective of this research is to examine the trends in the exchange rate markets of the ASEAN-5 countries (Indonesia (IDR), Malaysia (MYR), the Philippines (PHP), Singapore (SGD), and Thailand (THB)) through the application of dynamic moving average trading systems. This research offers evidence of the usefulness of the time-varying volatility technical analysis indicator, Adjustable Moving Average (AMA′) in deciphering trends in these ASEAN-5 exchange rate markets. This time-varying volatility factor, referred to as the Efficacy Ratio in this paper, is embedded in AMA′. The Efficacy Ratio adjusts the AMA′ to the prevailing market conditions by avoiding whipsaws (losses due, in part, to acting on wrong trading signals, which generally occur when there is no general direction in the market) in range trading and by entering early into new trends in trend trading. The efficacy of AMA′ is assessed against other popular moving-average rules. Based on the January 2005 to December 2014 dataset, our findings show that the moving averages and AMA′ are superior to the passive buy-and-hold strategy. Specifically, AMA′ outperforms the other models for the United States Dollar against PHP (USD/PHP) and USD/THB currency pairs. The results show that different length moving averages perform better in different periods for the five currencies. This is consistent with our hypothesis that a dynamic adjustable technical indicator is needed to cater for different periods in different markets.

## Introduction

Algorithmic trading has evolved exponentially in recent years and, along with it, interest in high-frequency trading has grown remarkably [1]. Accompanying this interest, there has been a number of studies of computational trading algorithms [2] that users find useful for investment timing [3]. These technical analysis elements are also relatively common in the foreign exchange markets [4], [5].

The problem confronting most financial market traders is how to differentiate a ranging market (when the price movements are confined between a lower boundary of support and an upper boundary of resistance) from a trending market when prices are steadily moving in a general upward or downward direction) [3]. It is important for the trader to correctly identify the market condition, as a ranging market requires technical analysis tools (such as leading momentum rate of change) that differ from those employed in a trending market (like lagging moving average). Identifying the wrong market condition and employing the wrong trading strategy can result in unnecessary losses known as whipsaws [3]. In an attempt to overcome this, our paper introduces a dynamic adjustable technical indicator, the Adjustable Moving Average′ (AMA′), to better time trading decisions in foreign exchange markets where studies show depleting abnormal returns in recent years [6], [7], [8], [9], [10]. AMA′ is intended to avoid some of these whipsaws and to allow early entry into new trends by employing a time-varying trend deciphering ratio called the Efficacy Ratio.

The research objectives of this study are twofold: i) to investigate the time-varying volatility characteristic of these five currencies (Indonesian rupiah (IDR), Malaysian Ringgit (MYR), Philippine Peso (PHP), Singapore Dollar (SGD), and Thai Baht (THB)); and ii) to assess the efficacy of moving averages, and in particular that of AMA′, to capture the dynamic nonlinear movements of these exchange rates in order to generate abnormal returns beyond those produced by the passive buy-and-hold, from 2005 to 2013 for the training in-sample period and 2014 for the out-of-sample period. The hypotheses tested in this study are that the returns from moving average technical rules generate significantly higher returns than the passive buy-and-hold, and that a dynamic time-varying indicator (like the adjustable AMA′) is more suitable than the popular and most optimized fixed length moving averages that are determined through hindsight. Apart from AMA′, this paper also evaluates the efficacy of eight technical trading rules, from the traditional simple moving averages used by Brock et al. [11] and Lukac et al. [12] to the dynamic AMA′ approach. The passive buy-and-hold strategy serves as the control. To increase the robustness of the findings, the results are also compared to those of optimized trading models. The findings show that these five ASEAN currencies exhibit time-varying volatilities throughout the sampling period. Consistent with earlier studies on currencies, the evidence supports the possibility of forecasting future price movements by analyzing only historical foreign exchange rates [6], [7], [8], [9]. The results of this study show that, after taking into account slippage costs (the differences between the theoretical execution prices, usually the last prices traded, and the actual prices executed), on the whole, the nine popular technical moving-average rules outperform the passive buy-and-hold in terms of abnormal returns. Furthermore, this study finds that the returns of the proposed AMA′ are comparable to those of the most optimized portfolio and can be used for trading in PHP and THB in the near future. More importantly, this study’s findings show that this new technical analysis indicator, AMA′, can be profitably employed in financial markets. The implications of our findings are i) from the perspective of financial market researchers, this study introduces a method of employing past volatilities to forecast the future prices, and ii) from the point of view of market practitioners, and especially of professional traders on the proprietary trading desks of financial institutions, AMA′ is ready to be implemented in their current trading strategies, especially if they are trading in United States Dollars against Philippines Peso (USD/PHP), or/and United States Dollars against Thai Baht (USD/THB).

This paper proceeds as follows. The next section presents a brief description of ASEAN-5 currencies and their daily returns. The third section describes the trading systems used. The fourth section discusses empirical findings. The final section concludes.

## Related Works

Allen and Taylor [6], Lee and Mathur [7], Levich and Thomas [8], Neely et al. [9], and Cheung and Wong [10] are amongst the seminal works evaluating the predictability power of technical trading rules in various foreign exchange markets. Levich and Thomas [8] opine that excessive speculation causes exchange rates to depart from their fundamental equilibrium for protracted periods of time. Other contributions involving more advanced techniques include those of Cheung and Wong [10], Gehrig and Menkhoff [5], Andrada and Fernandez [13], Zhang [14] and Jin and Kim [15].

Technical analysis is the forecasting of price movements by analyzing past market data [16]. It establishes specific trading rules using indicators, such as moving averages, to decipher behavioral patterns in time-series data [11], [12], [17], [18], [19], [20], [21], [22], [23]. The objective is to maximize profits while minimizing the risk of losses [19], [20]. The main rationale behind using the moving-average rule is that it provides a means of determining the general direction or trend of a market by smoothing out unnecessary noise [19]. This is especially meaningful for time-series prices, which are nonlinear because moving-average rules could capture information ignored by their linear counterparts [11], [19], [20], [21]. According to Brock et al. [11], the most popular moving-average rule is the 1–200 rule, in which the short period is one day and the long period is 200 days. Other common standards include the 1–50, 1–150, 5–200, and 2–200 rules [11], [12].

Even though the efficient market and random walk hypotheses contradict this approach by affirming that all public information in the market is immediately reflected in prices, and that abnormal returns can never be made with only knowledge of historical data [16], empirical evidence however, indicates that technical trading rules can produce abnormal profits in foreign exchange markets [7], [9]. Nevertheless, many have also highlighted the existence of a slow-moving downtrend for this kind of profitability over the last decade [24], [25], [26]. It can be seen that, as time passes, the direction of price changes converges to fit a geometric distribution [27]. To outperform the passive buy-and-hold in foreign exchange markets, increasingly complicated trading rules are needed [24], which might be a consequence of increasingly efficient market conditions in these markets [24], [25], [26], [27], [28], [29], [30]. If this were true, no profitable position could be gained from trading rules or technical analysis, since the prices of these markets would already reflect all relevant information [16].

Many studies have suggested that exchange rate volatility behaves non-monotonically [31] and that the market’s volatility can be used as an indicator of signs of maturation [30]. Despite this time-varying volatility element, most technical analyses have deployed simple moving average techniques in their estimations [11], [12],[21]. Black [32] finds that technical trading techniques are still lacking in accounting for the varying volatility clustering found in most financial time-series data. Gandolfi et al. [33] address time-varying volatilities in their study by employing an excess “volatility” technical indicator—the ratio of the 10-day standard deviation of closing prices to the 50-day standard deviation of closing prices—in order to determine the weights used in their innovative Moving Average Convergence Divergence (MACD) trading system. Noor et al. [34] employs a ratio of the 34-day standard deviation of closing prices to the 6-day standard deviation of closing prices to determine the length of the moving average used in their trading system, the Adjustable Bands Z-Test (ABZ′). Using the same concept, this paper introduces the Efficacy Ratio (the ratio of the most optimized parameter, n, divided by its square-root), to determine the appropriate length of AMA' suitable to the prevailing trend in different periods. The value of n is determined from the training in-sample period and employed in the out-of-sample period to determine the most suitable AMA's length to generate appropriate trading signal in a timely manner.

Unlike conventional methods, AMA′ automatically generates adaptive parameter to fit historical and current data. AMA′ captures a larger portion of the trend and, ultimately, greater abnormal profits by routinely adjusting the parameter according to prevailing market condition, whether ranging or trending. This is consistent with recent findings that statistical learning methods have produced better out-of-sample results than most single and fixed moving-average rules [3], [13].

## Data

The data used in this research are the exchange rate values of the currencies of the ASEAN-5 countries (Indonesian Rupiah (IDR), Malaysian Ringgit (MYR), Philippines Peso (PHP), Singapore Dollar (SGD), and Thailand Baht (THB)) quoted against the United States Dollar (USD)). These data were collected from the Bloomberg database for the period from January 2, 2005 to December 31, 2014. This period consists of two subperiods: i) for the training in-sample period from January 2, 2005 to December 31, 2013 and ii) for the out-of-sample period from January 2, 2014 to December 31, 2014. We selected these periods because we wanted to include extended periods of stable appreciation of these currencies from the beginning of 2005 to the turbulent period of volatile depreciation since the end of 2013. All the currencies are actively traded, so the problems associated with nonsynchronous trading should be of little concern.

Table 1 shows the statistical description of these currencies for each year from 2005 to 2013. From the different standard deviations recorded each year for each of the currencies, it can be seen that each displays time-varying volatilities.

**Table 1:** Descriptive statistics of the exchange rates of IDR, MYR, PHP, SGD, and THB against USD from 2005 to 2013 by the year.

	Full Sample	2004	2005	2006	2007	2008	2009	2010	2011	2012	2013
**IDR**											
Mean	9902	8938	9712	9166	9142	9694	10394	9082	8772	9388	10440
Median	9324	9036	9752	9145	9111	9285	10163	9040	8779	9454	9979
Maximum	14693	9440	10775	9815	9480	12650	12100	9428	9158	9799	12261
Minimum	8175	8317	9135	8703	8675	9060	9340	8890	8464	8888	9618
Std. Dev.	1416	335	346	168	171	906	854	138	207	229	842
**MYR**											
Mean	3.3827	NA	3.7703	3.6667	3.4361	3.3337	3.5241	3.2183	3.0585	3.0886	3.1505
Median	3.3235	NA	3.7701	3.6725	3.4465	3.2668	3.5243	3.1985	3.0403	3.0680	3.1591
Maximum	4.4570	NA	3.7810	3.7790	3.5300	3.6400	3.7280	3.4440	3.2048	3.2005	3.3346
Minimum	2.9390	NA	3.7463	3.5270	3.3115	3.1320	3.3605	3.0635	2.9390	2.9943	2.9625
Std. Dev.	0.2927	NA	0.0094	0.0514	0.0587	0.1484	0.0915	0.1055	0.0665	0.0518	0.0892
**PHP**											
Mean	46.97	56.05	55.04	51.27	46.14	44.49	47.63	45.08	43.30	42.22	42.47
Median	45.37	56.10	54.94	51.32	46.35	44.40	47.70	45.20	43.34	42.23	43.11
Maximum	56.46	56.46	56.36	53.52	49.14	49.94	49.03	47.13	44.59	44.12	44.73
Minimum	40.27	55.18	53.00	49.03	41.22	40.27	46.00	42.49	41.97	40.80	40.56
Std. Dev.	4.69	0.28	0.86	1.15	2.13	2.91	0.73	1.12	0.53	0.82	1.38
**SGD**											
Mean	1.4233	1.6900	1.6644	1.5887	1.5067	1.4146	1.4538	1.3625	1.2572	1.2494	1.2512
Median	1.3977	1.6954	1.6650	1.5838	1.5185	1.4124	1.4503	1.3741	1.2615	1.2508	1.2493
Maximum	1.7278	1.7278	1.7061	1.6605	1.5450	1.5302	1.5549	1.4241	1.3195	1.2971	1.2838
Minimum	1.2008	1.6314	1.6191	1.5344	1.4393	1.3482	1.3795	1.2822	1.2008	1.2163	1.2205
Std. Dev.	0.1567	0.0250	0.0229	0.0277	0.0324	0.0504	0.0471	0.0416	0.0313	0.0212	0.0148
**THB**											
Mean	34.23	56.05	55.04	51.27	46.14	44.49	47.63	45.08	43.30	42.22	42.47
Median	33.33	56.10	54.94	51.32	46.35	44.40	47.70	45.20	43.34	42.23	43.11
Maximum	42.14	56.46	56.36	53.52	49.14	49.94	49.03	47.13	44.59	44.12	44.73
Minimum	28.67	55.18	53.00	49.03	41.22	40.27	46.00	42.49	41.97	40.80	40.56
Std. Dev.	3.46	0.28	0.86	1.15	2.13	2.91	0.73	1.12	0.53	0.82	1.38

The daily spot exchange rates are converted into daily returns as follows:

$$R_{i}=ln\frac{C_{t}}{C_{t-1}}$$ (1.0)

where R_*i*_ is the return of the exchange rate of each currency, while C_*t*_ and C_*t-1*_ are the exchange rates determined at the close of days *t* and *t*-1, respectively. The summary statistics of the returns are shown in Table 2.

**Table 2:** Summary of descriptive statistics of the log first differenced daily returns series from 2005 to 2013.

	IDR	MYR	PHP	SGD	THB
**Mean**	-0.0133%	-0.0045%	0.0054%	0.0058%	0.0030%
**Median**	0.0000%	0.0000%	0.0093%	0.0082%	0.0000%
**Maximum**	6.4252%	3.5749%	1.8057%	2.3812%	3.1740%
**Minimum**	-4.4882%	-1.8679%	-1.6778%	-2.6702%	-3.8138%
**Std. Dev.**	0.0059	0.0043	0.0035	0.0035	0.0032
**Skewness**	0.0796	0.3930	-0.1290	0.0178	-0.5294
**Kurtosis**	17.1239	7.9856	4.5719	7.6945	19.3098
**Jarque–Bera**	27432.47	2887.07	329.24	2874.31	35317.07
**Probability**	0.00	0	0	0	0
**Sum**	-0.44	-0.122287	0.168349	0.181028	0.094477
**Sum Sq. Dev.**	0.11	0.049731	0.038183	0.037981	0.033337

Table 2 shows the statistical description of the five exchange rate returns for the in-sample period from January 2, 2005 to December 31, 2013. The results show that IDR and MYR have negative daily average returns of 0.01% and 0.004% respectively; PHP, SGD and THB show positive daily average returns of 0.0054%, 0.0058%, and 0.003% respectively. The daily movements of IDR and MYR are very volatile, as indicated by the large standard deviations of 0.0059 and 0.0045, compared with the smaller standard deviations of 0.0035, 0.0035, and 0.0032 recorded for PHP, SGD, and THB respectively. The IDR, MYR, and SGD distributions are positively skewed, while the PHP and THB distributions are negatively skewed. The kurtosis of the currencies’ returns indicates that all their distributions are leptokurtic. The Jarque–Bera test results confirm that the distributions of the daily returns of these currencies are not normal.

Table 2 shows that IDR and MYR, which depreciated the most against USD, have the highest and second highest volatilities of the ASEAN-5 currencies respectively. PHP, SGD, and THB, which remain relatively stable with slightly positive daily means, have smaller and quite similar standard deviations.

**Fig 1:**
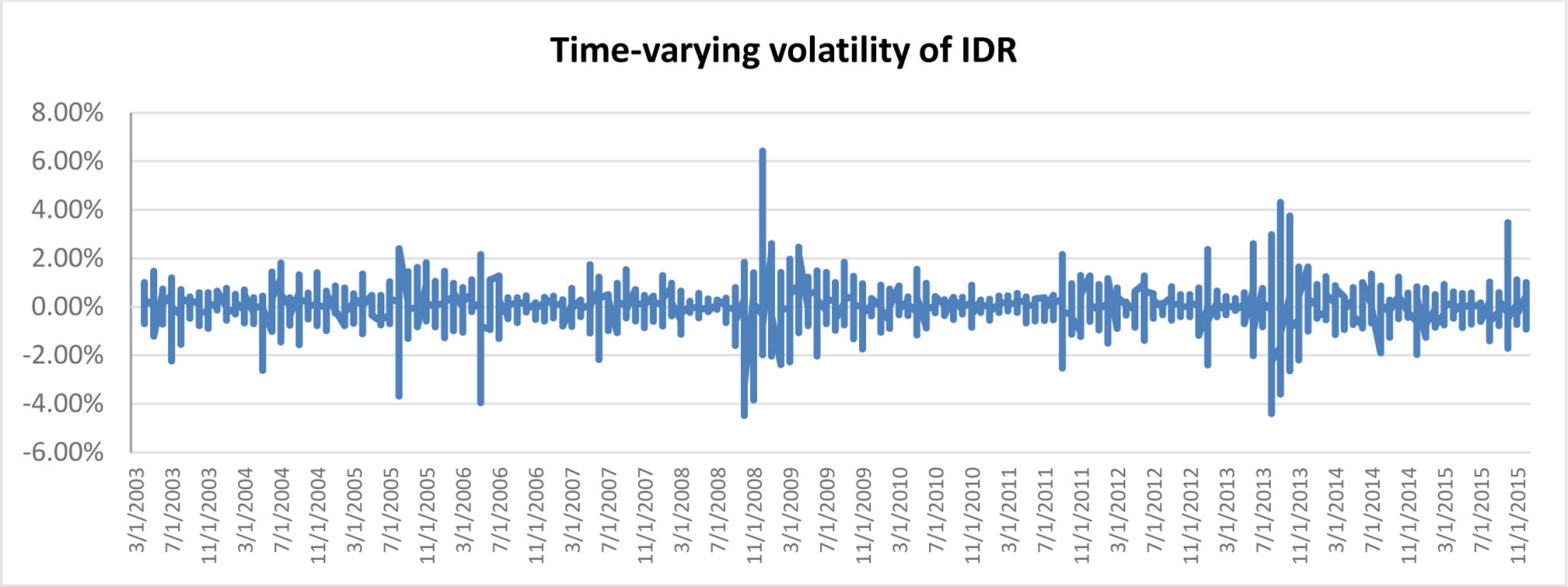
Time-varying volatilities of IDR from 2005 to 2013.

**Fig 2:**
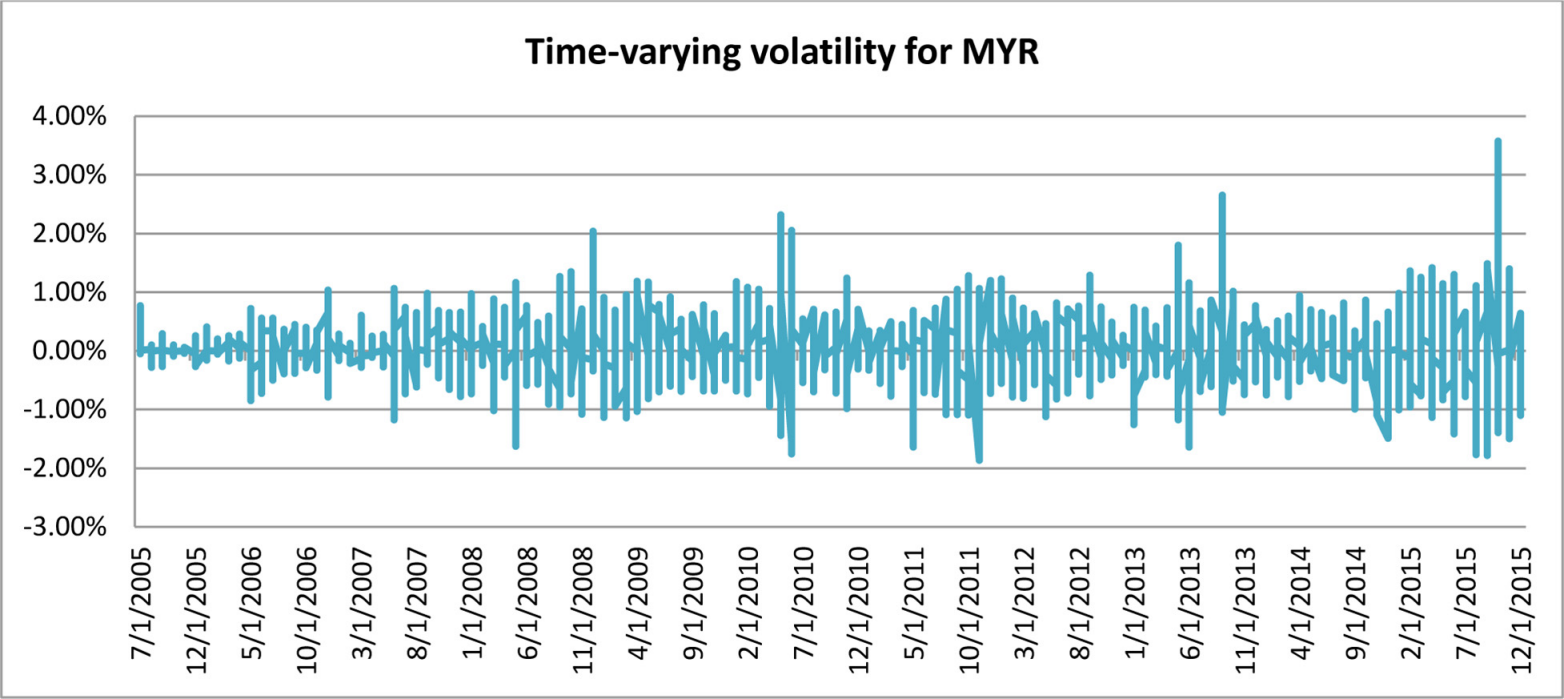
Time-varying volatilities of MYR from 2005 to 2013.

**Fig 3:**
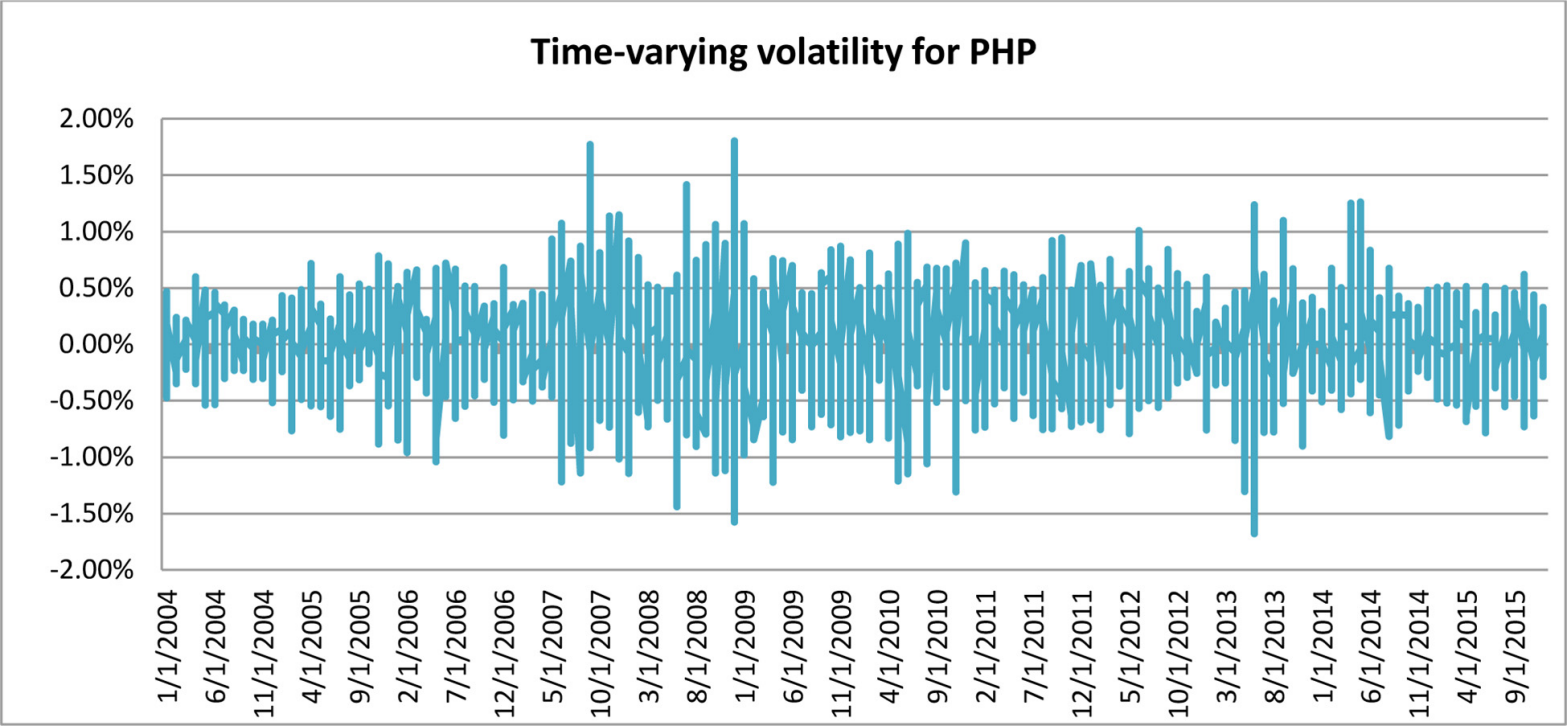
Time-varying volatilities of PHP from 2005 to 2013.

**Fig 4:**
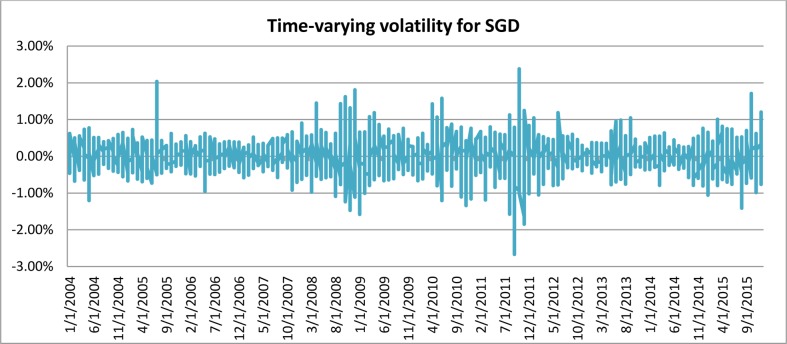
Time-varying volatilities of SGD from 2005 to 2013.

**Fig 5:**
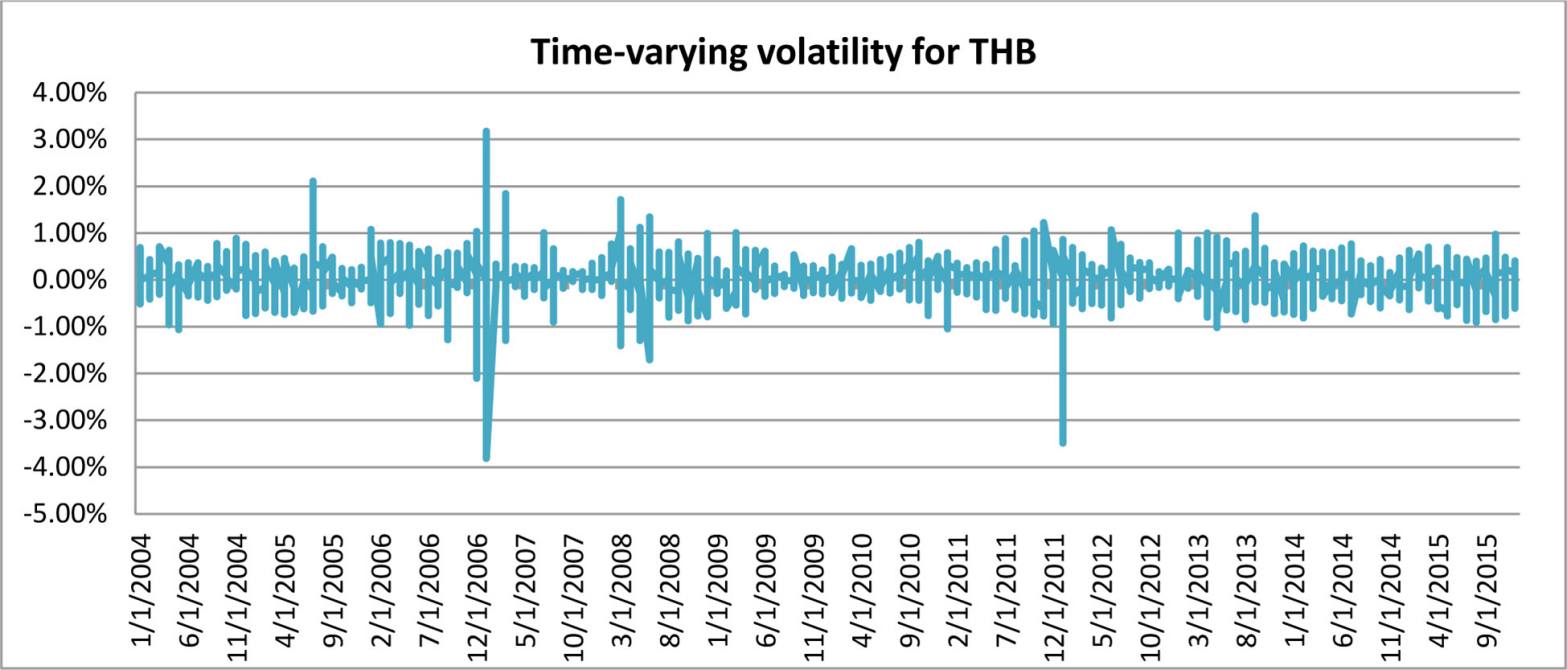
Time-varying volatilities of THB from 2005 to 2013.

Fig 1 depicts the time-varying volatilities of IDR while Figs 2–5 illustrate the time-varying volatilities characteristic of MYR, PHP, SGD, and THB respectively.

The figs show that the volatilities of each currency are dynamically changing from high volatility to small volatility, and vice versa. It can be seen from the figs above that the volatility element of each currency is dynamically changing all the time, and can be said to be time-varying.

IDR is the most volatile currency of the ASEAN-5 currencies. MYR is the second most volatile currency after being unpegged at 3.80 from July 22, 2005 to the present. It began strengthening by 11% in 2006, but has depreciated since then, at 9% the following year. It stayed close to 3.50 for most of 2008, 2009, and 2010. PHP spent most of 2006, 2007, and 2008 appreciating, and lost its gains in 2009. It then continued its appreciation in 2010, 2011, and 2012. It depreciated slightly in 2013 and 2014. SGD spent most of the 2006–2012 period appreciating before losing nearly all its gains in 2013 and 2014. THB’s pattern of appreciation and depreciation is very similar to SGD in this period.

IDR and MYR displayed high volatilities during the subprime period in 2008 and showed lower variations in other periods. PHP, THB, and SGD were relatively stable throughout the sampling period.

## Methods

The aim of this research is to ascertain whether one or more of the technical trading rules are superior to the passive buy-and-hold strategy, as advocated by Fama [16]. We thus employed the eight most commonly used technical indicators in the currencies market, as well as in the most popular and cited studies on technical analysis, namely Brock et al. [11] and Lukac et al. [12], alongside our proposed AMA′. Similar to these studies [11], [12], the passive buy-and-hold return is commonly used as the market benchmark. The eight most commonly used technical indicators found in Brock et al. [11] and Lukac et al. [12]; as well as in current foreign exchange market, and our proposed trading model are:

1. 5-day Simple Moving Average (SMA5);
2. 20-day Simple Moving Average (SMA20);
3. Moving Average Crossover (MAC);
4. Moving Average Convergence Divergence (MACD);
5. Kaufman Adaptive Moving Average (KAMA);
6. 1% Bands from 20-day Moving Average (MA20,1%);
7. The most optimized moving averages on hindsight (Opt MA_p_) for the portfolio of these five currencies (as an ideal benchmark);
8. The optimized moving average on hindsight (Opt Ma_c_) for each individual currency (as comparisons of ideal benchmarks); and
9. AMA′.

A trading model is regarded as ideal if it meets the following criteria:

1. it should not produce very large losses or exhibit any net large losses in any year;
2. the model should work well both in the testing stage and in practice, and it should automatically adjust to shifts in parameters; and
3. it must produce abnormal returns even after accounting for transaction and slippage costs.

This study thus adopts a similar testing approach based on the technical trading rules specified by Brock et al. [11] and other innovations using moving averages, which include variable-length and fixed-length moving-average rules [35], [36].

The following notes summarize the nine methods used in the back-tests:

### 5-day Simple Moving Averages (SMA5)

The 5-day simple moving average used by Brock et al. [11] and Lukac et al. [12] is a popular and simple mechanical trend trading system. This moving average is referred to as SMA (C,5,0%), where C represents the closing price, 5 is the five-period moving average, and 0% refers to 0% from the simple moving average. The moving average is computed as follows:

$${SMA}_{n_{t}}=\frac{1}{n}\sum_{i=0}^{n}C_{t-1}$$ (2.0)

where SMA is simple moving average, *n* is 5 days, and *C*_*t*_ is the closing price at period *t* for each currency (USD/local currency). Five days is selected as the period as there are five trading days in a week. When C_t_ > SMA5_*t*_, we buy USD (selling the corresponding ASEAN-5 currency); otherwise, we sell USD.

### 20-day Simple Moving Averages (SMA20)

The most popular simple technical indicator used in the foreign exchange market is the 20-day simple moving average [11], [12]. This variable moving average is referred to as SMA (C,20,0%), where C represents the closing price, 20 is the 20-period moving average, and 0% refers to 0% from the simple moving average. The 20-day moving average is popularly used and quoted as there are 20 trading days in a month.

When C_t_ > SMA20_*t*_, we buy USD (selling the corresponding ASEAN-5 currency); otherwise, we sell USD.

### 3-day and 21-day Moving Averages Crossover (MAC 3,21)

Similar to Brock et al. [11], we apply the moving averages crossover, also known as the Variable Moving Average (3,21,0%), where 3 refers to the 3-day moving average period, 21 refers to 21-day moving period and 0% refers to 0% from the averages. A buying signal is generated when SMA3_t_ > SMA21_t_; a selling signal otherwise. The moving-average lengths of 3 and 21 days are most commonly used by market practitioners (as indicated by the default parameter setting in Bloomberg) for the moving average crossover.

### Moving Average Convergence Divergence (MACD)

Since its introduction by Appel [35], Moving Average Convergence Divergence (MACD) has become one of the most popular technical indicators used and quoted, even though many retail investors do not know how to calculate it. This technical indicator subtracts a longer-term exponential moving average (EMA_lt_) from a shorter-term exponential moving average (EMA_st_). MACD_t_ is calculated as follows:

$${MACD}_{t}={EMA}_{st}{-EMA}_{lt}$$ (3.0)

where

$$EMA_{st}\;is\;[C_{t}-{EMA}_{t-1}]x\;\left[\frac{2}{st+1}\right]+\;{EMA}_{t-1}\;where\;st\;represents\;12\text{-}day\;moving\;average.$$ (3.1)

$$EMA_{lt}\;is\;[C_{t}-{EMA}_{t-1}]x\;\left[\frac{2}{lt+1}\right]+\;{EMA}_{t-1}\;where\;lt\;refers\;to\;26\text{-}day\;moving\;average.$$ (3.2)

When MACD > Trigger Line, a buy signal is generated; otherwise, a sell signal is generated.

$$The\;Trigger\;Line\;is\;computed\;as:\;[C_{t}-{MACD}_{t-1}]x\;\left[\frac{2}{9+1}\right]+\;{MACD}_{t-1}$$ (3.3)

or by taking the 9-day exponential average of MACD.

### Kaufman Adaptive Moving Average (KAMA)

To vary the moving average according to market conditions, Kaufman [36] allots different weights to current data and past smoothened data, using an Efficiency Ratio (ER), as reflected in the following equation:

$${KAMA}_{t}={\alpha \;ER}_{t}{+(1-\alpha ER}_{t}){KAMA}_{t-1}$$ (4.0)

where:

$$\alpha =[(ER\;({\frac{2}{3}-\frac{2}{31}))+\frac{2}{31}]}^{2}$$ (4.1)

$$and\;ER_{t}=\frac{\left(C_{t}-C_{t-1}\right)}{\sum \;\left|C_{t}-C_{t-1}\right|}$$ (4.2)

in which *C*_*t*_ denotes the most current close and *C*_*t-1*_ is the previous close. If C_*t*_ > KAMA_*t*_, the signal is to buy; otherwise, the signal is to sell.

### Moving Average Envelope Band (SMA (1,20,1%))

To overcome the complications resulting from whipsaws in a ranging market, a certain percentage band above and below the moving average is added. In Brock et al. [11], this technical trading rule is referred to as SMA(1,20,1%), where a 1% band is constructed above and below the 20-day simple moving average. To construct the upper band, 1% is added to the 20-day simple moving average; to establish the lower band, 1% is subtracted from the 20-day moving average. The level 1% above the moving average gives additional confirmation of an uptrend, while the 1% lower mark confirms the downtrend. A buy-on-uptrend signal is called upon when the closing price crosses the upper 1% band. An exit-long signal is triggered when the closing price returns below the upper 1% band. Similarly, a sell-on-downtrend signal will be prompted when the closing price dips below the lower 1% band, and an exit-short signal will be generated when the price bounces back above the lower 1% band.

The upper 1% band is calculated as follows:

$${Upper\;1\%\;Band=1.01\times SMA}_{nt}$$ (5.1)

Similarly, the lower 1% band is calculated as follows:

$${Lower\;1\%\;Band=0.99\times SMA}_{nt}$$ (5.2)

where ${SMA}_{n_{t}}=\frac{1}{n}\sum_{i=0}^{n}C_{t-1}$.

### Benchmark Model: The Most Optimized Simple Moving Average (OptMA) for the portfolio of ASEAN-5 currencies

To maintain robustness in our technical analysis performance evaluation, besides benchmarking against the passive buy-and-hold strategy, we also compared the above trading rules with the most optimized parameter of a simple moving average based on historical performance on the portfolio of ASEAN-5 currencies. While only guesswork can suggest most optimized parameter in future, from the back-tests conducted in hindsight, 19 is the most optimized parameter within the range of 1 to 200 days for the combined portfolio of these ASEAN-5 currencies for this given in-sample period.

### Benchmark Models: The Optimized Simple Moving Average (OptMA) for each of ASEAN-5 currencies

As can be seen in Table 2, the different currency returns have different volatilities. Additional optimization exercises are thus conducted for each individual currency separately during the in-sample period, and the most optimized moving average for IDR is confirmed to be 19; and 22, 3, 12, and 3 for MYR, PHP, SGD, and THB, respectively. It can be seen that longer length moving averages are more suitable for IDR and MYR, which recorded the highest volatilities; the shorter moving averages fit the more stable and predictable PHP, SGD, and THB.

### Adjustable Moving Average′ (AMA′)

Studies have shown the existence of time-varying volatility in financial and economic time-series data [27], [30], [31]. It can also be seen from Table 1, as well as from Figs 1–5, that each different currency experiences different volatility in different years. Following this observation, the Adjustable Moving Average′ (AMA′) could prove useful, as it is capable of adjusting the trading rule in response to prevailing volatility condition. While Gandolfi et al. [33] use the excess volatility ratio and KAMA uses the Efficiency Ratio to allocate the weights of current and past smoothened data [36], AMA′ changes each period length of the moving average according to the prevailing Efficacy Ratio. In this paper, AMA′ uses a ratio of long-term standard deviation over short-term standard deviation. This new indicator is referred to as the Efficacy Ratio (*v*_t_). The technical indicator called the Adjustable Moving Average (AMA′) is then determined as follows:

$${AMA\prime }_{t}=\frac{1}{\nu_{t}}\sum_{i=0}^{\nu }C_{t-1}$$ (6.0)

where *v*_*t*_ is the Efficacy Ratio and $v_{t}=\frac{\sigma_{lt}}{\sigma_{st}}$ (σ_lt_ is the long-term standard deviation, σ_st_ is the short-term standard deviation).

If C_*t*_ > AMA′_*t*_, then the signal is to buy USD. If C_*t*_ < AMA′_*t*_, then the signal is to sell USD. This Efficacy Ratio (Eq 6.0) is a time-varying parameter that is automatically adjusted according to the current market condition. The Efficacy Ratio generates a longer moving average length when the market moves within a certain trading range for a continuous period of time. It generates a shorter length when the closing prices are trending. This tests the hypothesis that different market conditions require dynamic moving averages to generate appropriate trading signals.

**Fig 6:**
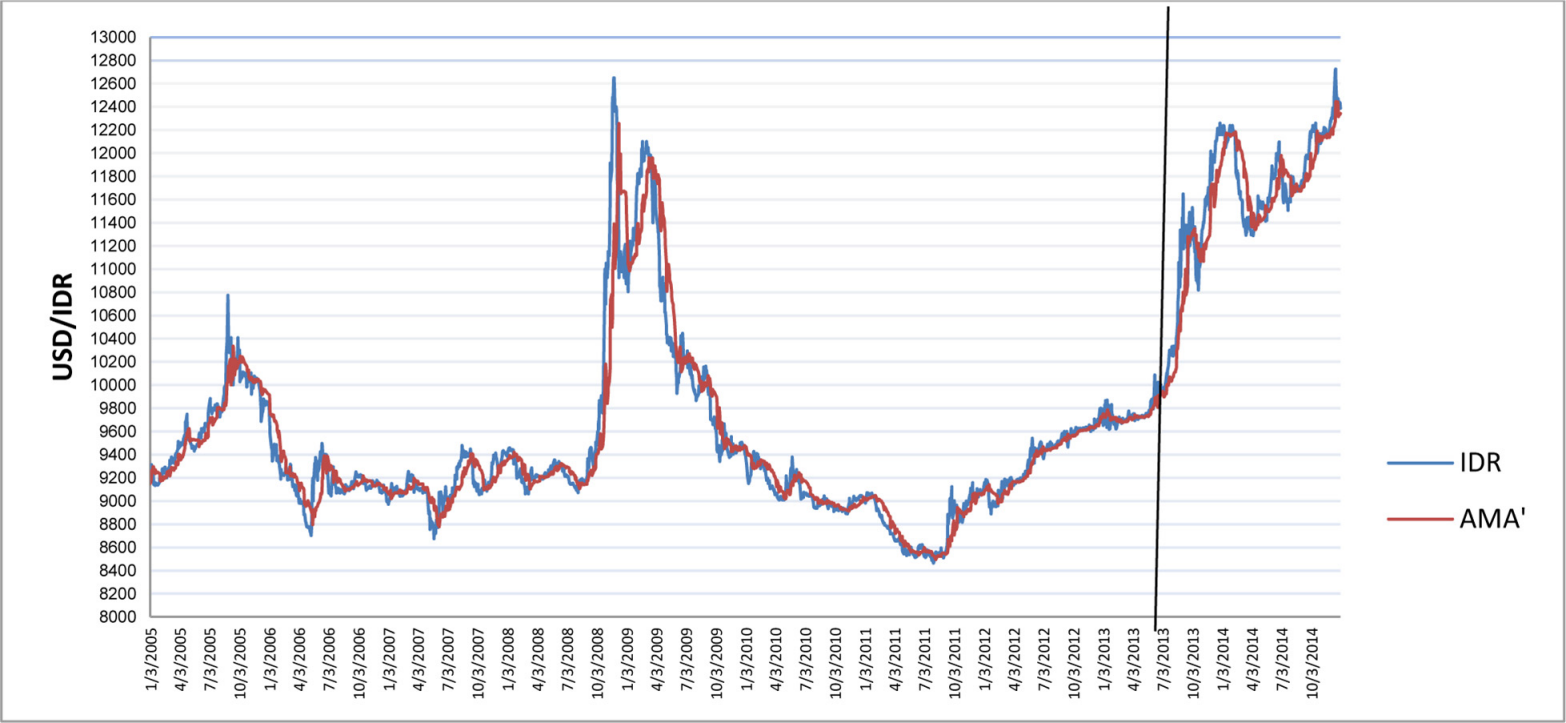
Chart of USD/IDR with AMA′ from 2005 to 2013.

**Fig 7:**
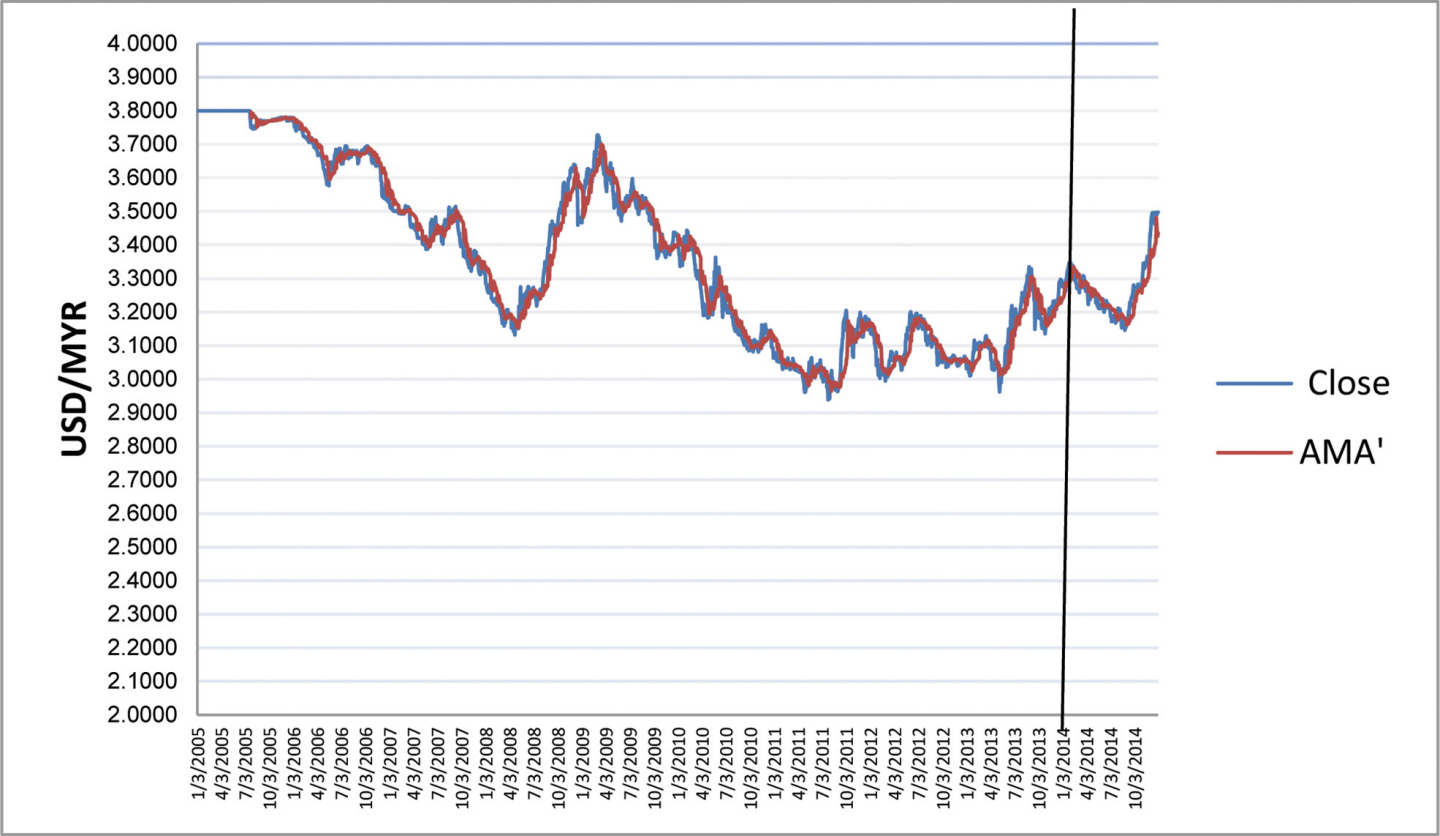
Chart of USD/MYR with AMA′ from 2005 to 2013.

**Fig 8:**
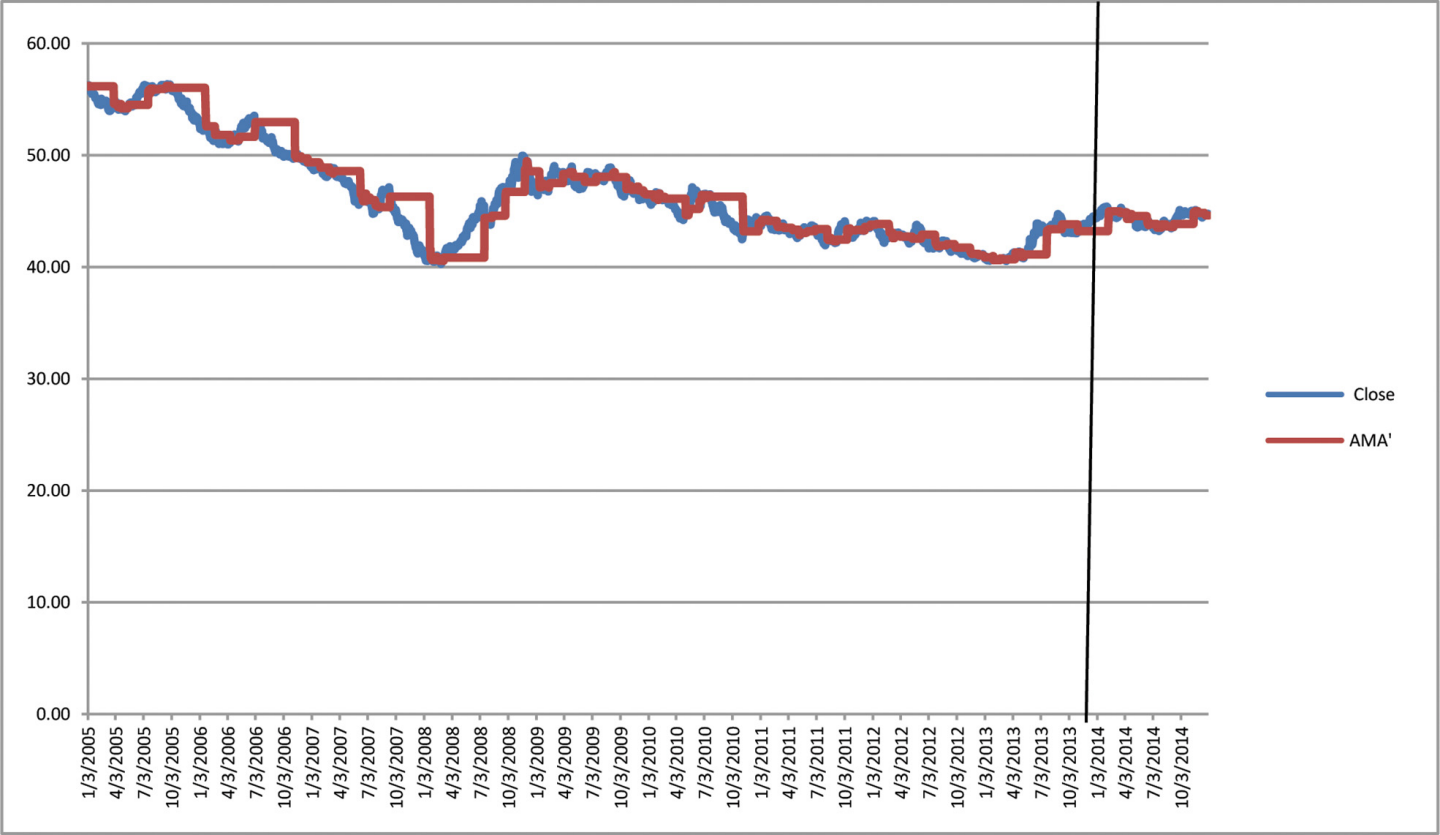
Chart of USD/PHP with AMA′ from 2005 to 2013.

**Fig 9:**
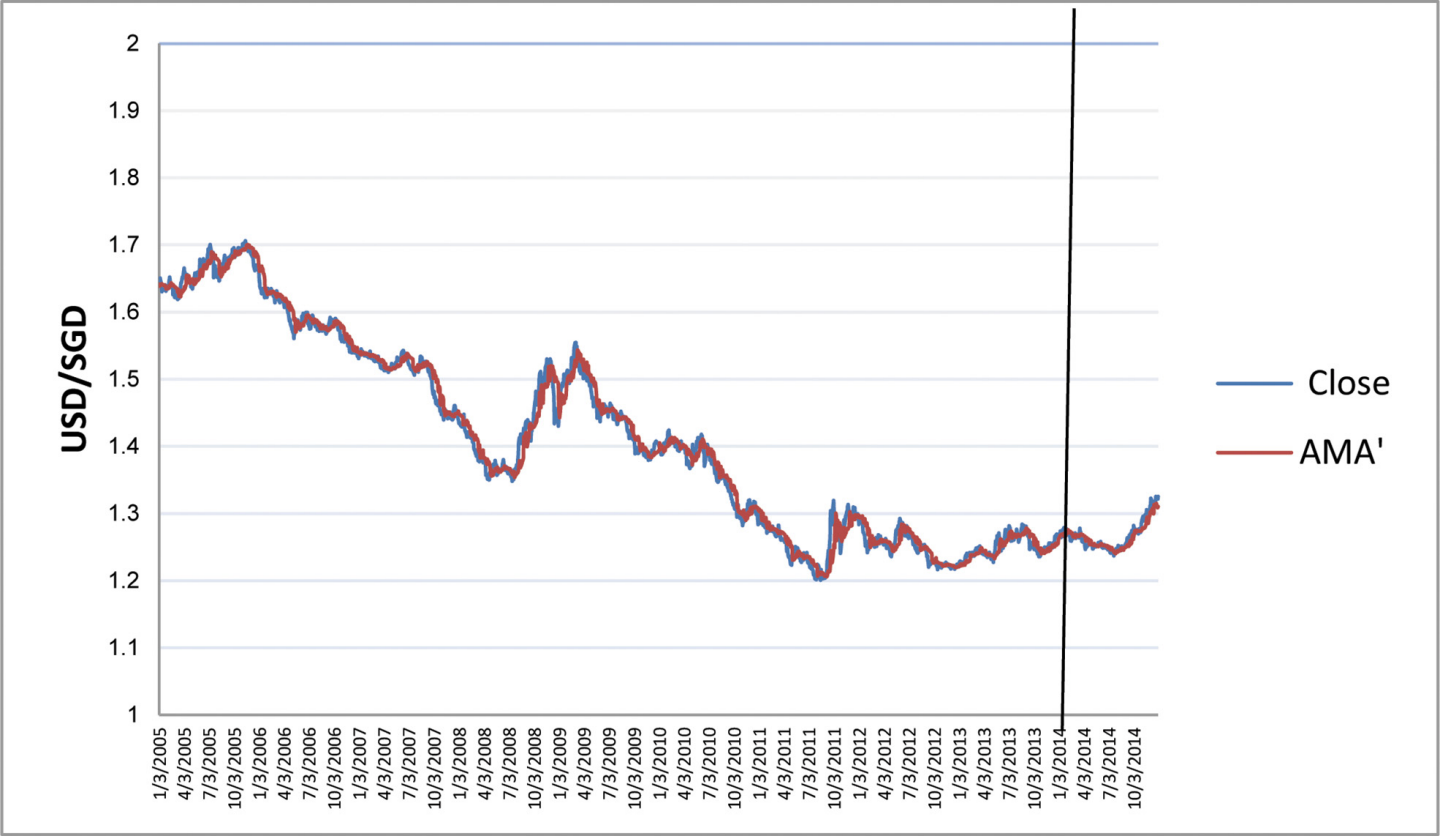
Chart of USD/SGD with AMA′ from 2005 to 2013.

**Fig 10:**
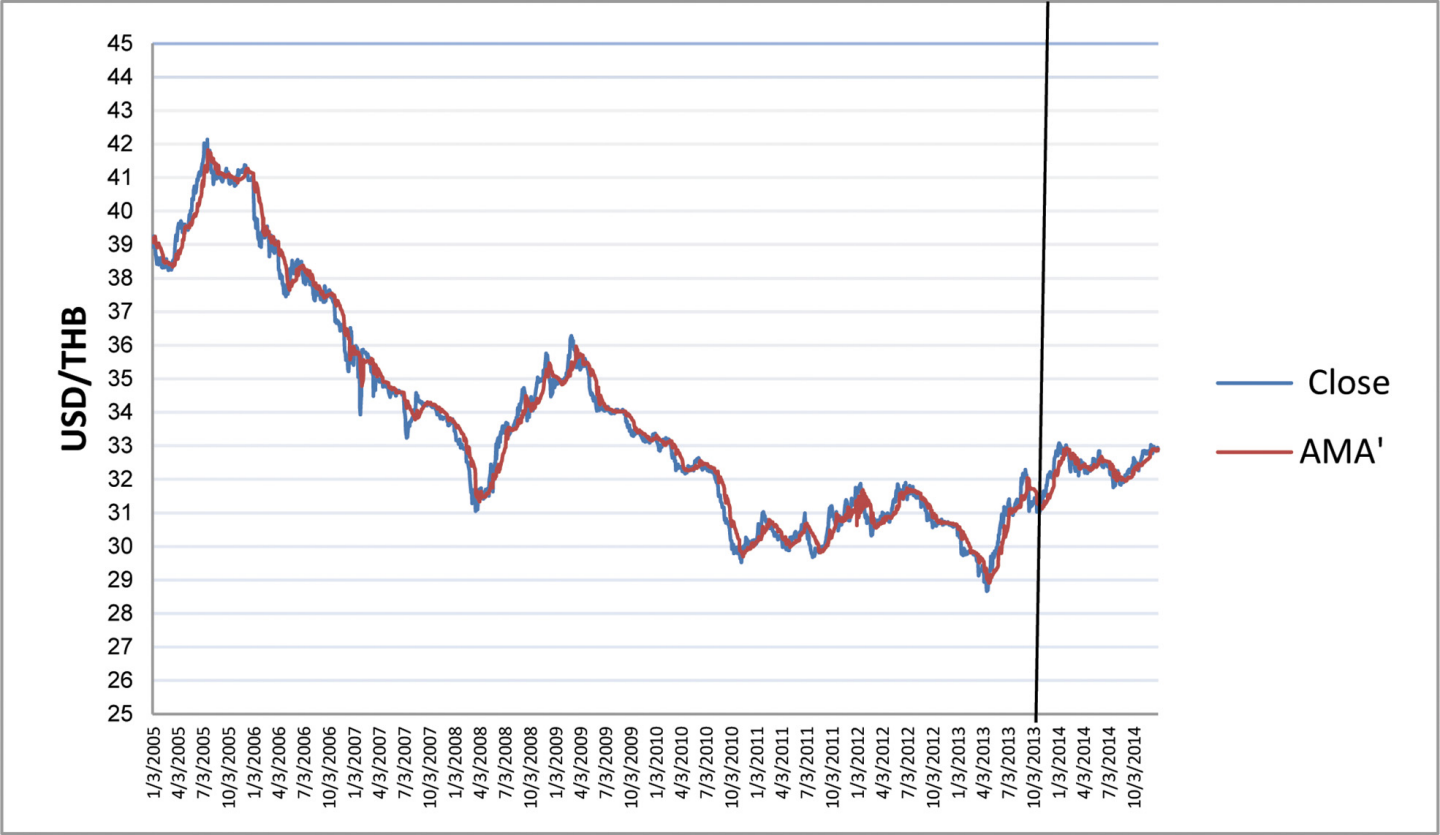
Chart of USD/THB with AMA′ from 2005 to 2013.

The charts in Figs 6–10 depict IDR, MYR, PHP, SGD, and THB respectively against USD, along with the corresponding AMA′ are as follows:

Note that AMA′_*t*_ adjusts and follows to the underlying currency accordingly. We use the nine estimation methods to test the hypothesis that, in the long run, mechanical trading rules generate significantly higher returns than buy-and-hold, that is:

H1: Mechanical trading rules generate excess return compared to buy-and-hold.

As foreign exchange does not incur transaction costs, to account for possible slippages (when the order is executed at a price worse than the last traded price), this study adds one tick (minimum fluctuation) for slippage. Slippage is a real issue in actual trading.

## Results and Discussion

The results show that, on the whole, even after taking slippage costs into account, the eight popular technical moving-average rules in the market and our proposed AMA′ generate significantly higher returns than the passive buy-and-hold, while the AMA′ generates significantly higher returns than the other six moving-average rules and is comparable to the portfolio’s optimized 19-day Moving Average and the individual currency’s optimized Moving Average. This follows and is consistent with the observation of time-varying volatilities in the ASEAN-5 currencies.

Table 3 shows the test results for buy-and-hold and the nine mechanical trading rules for the ASEAN-5 currencies in the training in-sample period from 2005 to 2013. The results show that all nine trading rules performed better than the passive strategy for MYR, PHP, SGD and THB, even after considering and adjusting 1 tick per transaction for the slippage cost. For IDR, all mechanical trading rules except AMA′ generated higher returns than the benchmark strategy.

**Table 3:** Buy-and-hold returns versus mechanical trading rules (SMA5, SMA20, MAC, MACD, KAMA, MA20,%, Portfolio’s OptMA19, Different Currencies OptMa, and AMA′): Returns for IDR, MYR, PHP, SGD, and THB from 2005 to 2013 taking into account slippage costs of 1 tick per transaction.

In-Sample Period										
*RETURNS*	*BH*	*SMA5*	*SMA20*	*MAC*	*MACD*	*KAMA*	*MA20*,*1%*	*Opt MA19*	*Most OptMA*	*AMA′)*
**IDR**	3383	6538	9260	8412	6387	6802	4732	9352	9352	2050
***P-Value***	* *	*0*.*276*	*0*.*141*	*0*.*168*	*0*.*281*	*0*.*255*	*0*.*392*	*0*.*143*	*0*.*143*	*0*.*425*
**%**	40.18%	77.65%	109.98%	99.90%	75.86%	80.78%	56.20%	**111.07%**	**111.07%**	24.35%
**MYR**	-0.4668	-0.3246	0.6202	0.6966	0.2624	-0.3554	-0.0719	0.6017	0.9606	1.2527
***P-Value***		*0*.*432*	*0*.*114*	*0*.*091*	*0*.*152*	*0*.*447*	*0*.*288*	*0*.*104*	*0*.*061*	***0*.*022***
**%**	-12.28%	-8.54%	16.32%	18.33%	6.91%	-9.35%	-1.89%	15.83%	25.28%	**32.97%**
**PHP**	-11.91	7.56	15.68	11.97	10.62	5.71	2.86	14.64	11.08	16.95
***P-Value***		*0*.*100*	***0*.*043***	*0*.*063*	*0*.*066*	*0*.*117*	*0*.*153*	***0*.*047***	*0*.*065*	***0*.*043***
**% **	-21.20%	13.46%	27.91%	21.31%	18.91%	10.16%	5.09%	26.06%	19.72%	**30.18%**
**SGD**	-0.3697	-0.0327	0.082	0.246	0.1869	-0.0757	0.0546	0.0879	0.2735	0.212
***P-Value***		*0*.*081*	*0*.*062*	***0*.*025***	***0*.*023***	*0*.*157*	***0*.*039***	*0*.*058*	***0*.*009***	***0*.*029***
**%**	-21.75%	-1.92%	4.82%	14.47%	10.99%	-4.45%	3.21%	5.17%	**16.09%**	12.47%
**THB**	-8.1789	6.39	10.56	13.71	4.23	8.95	2.85	13.39	10.66	17.62
***P-Value***	* *	*0*.*085*	***0*.*036***	***0*.*015***	*0*.*097*	***0*.*041***	*0*.*102*	***0*.*018***	***0*.*024***	***0*.*005***
**%**	-20.65%	16.14%	26.67%	34.62%	10.68%	22.60%	7.20%	33.81%	26.92%	**44.49%**

The most optimized simple moving average parameter between 1 to 200 in the back-test that generated the most profit for the portfolio of these five currencies is the 19-day simple moving average (traders generally usually use a 20-day or 21-day simple moving average, as there are about 20 or 21 trading days in a month). The Optimized SMA19 seems to best cater to IDR’s volatility, while AMA′ adjusts itself to suit MYR, PHP, and THB.

The *t*-test is performed to confirm *H1*. Based on the results, we find that THB records the greatest profit for five trading rules (SMA20, MAC, KAMA, OptMA19 and AMA′) against the conservative buy-and-hold. Four trading rules (MAC, MACD, MA20,1% and OptMA19) produce greater profits than the conservative strategy for SGD. SMA20, OptMA19 and AMA′ outperforms buy-and-hold for PHP. Only AMA′ outperforms buy-and-hold for MYR. In contrast to these results, none of the trading rules could significantly outperform the conservative buy-and-hold strategy for IDR. The *t*-test statistics show that the returns of AMA′ are significantly higher than those of buy-and-hold for MYR, PHP and THB. The most optimized n and √n, as the long-term and short-term standard deviations for MYR, PHP and THB, are 18, 32 and 25 respectively.

In summary, the results of the *t*-test show that technical trading rules like AMA′ are better at generating profits, as their parameters can be adjusted to the time-varying volatilities of the ASEAN-5 currencies other than IDR.

Table 4 presents the out-of-sample returns from buy-and-hold and the nine mechanical trading rules for the ASEAN-5 currencies for the period from January 2, 2014 to December 31, 2014. The purpose of testing the out-of-sample returns is to validate the future use of optimized trading parameters for optimized moving averages and AMA′. The returns of the optimized trading rules are generated using the most optimized parameters learnt during the training in-sample period (from January 2, 2005 to December 31, 2013) for simple moving average and AMA′ obtained in the same training in-sample period. The out-of-sample results for PHP and THB validate the use of AMA′ employing 32 and 25 respectively as the most optimized n. AMA′ generated the greatest return for IDR in 2014. Buy-and-hold outperforms all nine mechanical trading rules for MYR and SGD for 2014.

**Table 4:** Buy and hold returns versus mechanical trading Rules (SMA5, SMA20, MAC, MACD, KAMA, MA20,%, Portfolio’s OptMA19, Different Currencies OptMa, and AMA′): Returns for IDR, MYR, PHP, SGD, and THB from January 2, 2014 to December 31, 2014 after taking into account slippage costs of 1 tick per transaction.

Out-of-Sample Period										
*PROFIT*	*BH*	*SMA5*	*SMA20*	*MAC*	*MACD*	*KAMA*	*MA20*,*1%*	*Opt MA19*	*Most OptMA (19)*	*AMA′ (22)*
**IDR**	193	1015	519	946	932	958	658	593	593	1817
***%***	*1*.*97%*	*10*.*36%*	*5*.*30%*	*9*.*66%*	*9*.*52%*	*9*.*78%*	*6*.*72%*	*6*.*06%*	*6*.*06%*	***18*.*55%***
**MYR**	0.2216	0.0484	0.079	0.0278	-0.1025	0.1845	0.0269	0.1146	0.1288	0.1312
***%***	***7*.*25%***	*1*.*58%*	*2*.*58%*	*0*.*91%*	*-3*.*35%*	*6*.*03%*	*0*.*88%*	*3*.*75%*	*4*.*21%*	*4*.*29%*
**PHP**	0.32	-0.11	-0.16	0.67	-0.48	-1.92	-0.32	-0.21	-2.17	0.75
***%***	*0*.*78%*	*-0*.*27%*	*-0*.*39%*	*1*.*63%*	*-1*.*17%*	*-4*.*68%*	*-0*.*78%*	*-0*.*51%*	*-5*.*29%*	***1*.*83%***
**SGD**	0.0625	-0.0411	0.0043	0.0109	-0.0474	0.0156	-0.0285	0.0018	0.014	-0.0178
***%***	***5*.*12%***	*-3*.*36%*	*0*.*35%*	*0*.*89%*	*-3*.*88%*	*1*.*28%*	*-2*.*33%*	*0*.*15%*	*1*.*15%*	*-1*.*46%*
**THB**	0.19	-0.13	-1.32	-1.3	-1.51	-0.75	-0.39	-1.32	-0.32	1.26
***%***	*0*.*62%*	*-0*.*42%*	*-4*.*32%*	*-4*.*25%*	*-4*.*94%*	*-2*.*45%*	*-1*.*27%*	*-4*.*32%*	*-1*.*05%*	***4*.*12%***

Table 5 presents the correlation estimations between the volatilities of the currencies and the profits of the trading rules. The results show that most of the correlations are positive, except for KAMA(PHP) and MACD(SGD). In line with risk and return theory, the findings suggest that when the exchange rates are volatile, technical traders are able to generate higher returns. Therefore, traders should be prepared to tolerate higher risks in volatile markets for higher returns.

**Table 5:** Correlations between the five ASEAN currency returns and the mechanical trading rule returns (SMA5, SMA20, MAC, MACD, KAMA, MA20,%, Portfolio’s OptMA19, Different Currencies OptMa, and AMA′) from 2005 to 2013 after taking into account slippage costs of 1 tick per transaction

	BH	SMA5	SMA20	MAC	MACD	KAMA	MA20,1%	Opt MA19	Most OptMA (19)	AMA′ (22)
**IDR**	0.29	0.67	0.88	0.88	0.66	0.72	0.86	0.89	0.89	0.07
**MYR**	0.63	0.58	0.38	0.60	0.33	0.33	0.60	0.40	0.63	0.40
**PHP**	0.76	0.21	0.08	0.19	0.59	-0.04	0.44	0.06	0.13	0.04
**SGD**	0.60	0.42	0.52	0.42	-0.39	0.34	0.33	0.47	0.50	0.54
**THB**	0.54	0.44	0.73	0.68	0.35	0.31	0.55	0.59	0.23	0.32

As shown in Table 6, the annual mean returns (the average of net return after slippage costs) using the mechanical technical rules outperform the buy-and-hold for all the ASEAN-5 currencies for the entire period under review from 2005 to 2014. This supports our hypothesis that mechanical technical rules can generate higher returns than the passive threshold buy-and-hold advocated by Fama [16].

**Table 6:** Buy-and-hold annual mean returns and mechanical trading rules (MAC, MACD, KAMA, MA20,%, Portfolio’s OptMA19, Different Currencies OptMa, and AMA′): Annual mean returns for IDR, MYR, PHP, SGD, and THB from 2005 to 2014 after taking into account slippage costs of 1 tick per transaction.

Currency	Buy and Hold	Best Technical Indicator	Opt SMA19	Opt SMA	AMA′
IDR	4.21%	10.96%	11.71%	11.71%	4.29%
MYR	-0.50%	2.44%	1.96%	2.95%	3.73%
PHP	-2.04%	2.29%	2.55%	1.44%	3.20%
SGD	-1.66%	1.57%	0.53%	1.72%	1.10%
THB	-2.00%	3.04%	2.95%	2.59%	4.86%
Total	-2.00%	20.30%	19.71%	20.42%	17.18%

Even though IDR depreciated while the other four ASEAN currencies appreciated against USD during this period from 2005 to 2014, the annual gain from holding this portfolio of currencies is -2%, whereas the annual gains from trading these currencies using mechanical technical rules OptSMA19, MAC or KAMA, OptSMA_c_, and AMA′ are 20.42%, 20.30%, 19.71% and 17.18% respectively. The best technical indicator for IDR, MYR, PHP and SGD is MAC, while for THB, KAMA is best. By optimizing past historical data, we can find the most suitable mechanical technical rule and the most profitable parameter to employ so as to obtain the highest return. For the out-of-sample period, we can only validate that AMA′ with 32 for its long-term standard deviation continues to generate the highest return for PHP, while AMA′ with 25 for its long-term standard deviation can be used for THB.

This study has thus found some evidence that the random walk hypothesis, as advocated by Fama [16], may not fully explain the abnormal returns that can be made with technical trading rules, like moving averages, which are based on historical data. This evidence supports the earlier finding of Neely et al. [9] that technical trading rules can produce abnormal profits for foreign exchange markets. This is also consistent with the findings from Brock et al. [11], Lukac et al. [12], Irwin and Park [37], and Sullivan et al. [38] that a moving average generates higher returns than the buy-and-hold and that, in recent years, foreign exchange trading can be profitable using more innovatively adaptive trading systems [13], [24]. The finding that different length moving averages are suitable for different currencies for different periods also supports the finding of Gondolfi et al. [33]. For IDR, in the out-of-sample period, Optimized SMA19 offers the best return; for MYR, PHP, and THB, AMA′ offers the highest returns.

## Conclusion

Using the five ASEAN currencies for the period 2005 to 2014, this study assesses the efficacy of nine technical trading rules, including AMA′, against buy-and-hold. The results show that, except for the single case of AMA′ not being able to outperform buy-and-hold during the in-sample period for IDR, all the trading models are able to outperform the passive buy-and-hold strategy as they are able to capture abnormal returns on downtrends as well as uptrends found in these ASEAN-5 currencies. AMA′ generates the highest annual mean returns for MYR, PHP and THB while Opt SMA10 and Opt SMA 12 produce the most annual mean returns for IDR and SGD respectively. Each of the ASEAN-5 currencies has displayed inherent distinct volatility characteristic in different period. Thus different length moving average is required to cater to the different market condition. This finding is consistent with the studies conducted by Brock et al. [11], Lukac et al. [12], Andrada-Felix et al. [13], Kwon and Kish [21], and Sullivan et al. [38]. While simple moving-average rules like OptSMA19 and Opt SMA*c* outperformed the other technical models ex-post, ex-ante it is extremely difficult to estimate accurately the optimal lengths to be deployed [33]. Without the benefit of hindsight, AMA′ is able to learn from the behavior of past volatilities and yields and shows the most promising returns for PHP and THB in both the training in sample and the out-of-sample periods, regardless of slippage, by automatically adjusting the length of its moving average to suit the current trends prevailing in these markets.

Overall, this paper has demonstrated the efficacy of technical trading models in the ASEAN-5 currency markets. Whilst ex-post simple moving averages are better than the passive strategy in hindsight, an ex-ante time-varying volatility technique such as the AMA′ can be comparatively superior, given its ability to produce abnormal returns in different periods and markets.

For researchers and academics, as well as for market practitioners and especially for the algorithmic trading desks of large financial institutions, AMA′s ability to profitably adjust to time-varying volatilities, as demonstrated in this article, points to a new research direction for mechanical learning trading systems and could eventually be adopted as a professional trading strategy.
